# Network Challenges for Cyber Physical Systems with Tiny Wireless Devices: A Case Study on Reliable Pipeline Condition Monitoring

**DOI:** 10.3390/s150407172

**Published:** 2015-03-25

**Authors:** Salman Ali, Saad Bin Qaisar, Husnain Saeed, Muhammad Farhan Khan, Muhammad Naeem, Alagan Anpalagan

**Affiliations:** 1 School of Electrical Engineering and Computer Science (SEECS), National University of Sciences and Technology (NUST), Islamabad, 44000, Pakistan; E-Mails: saad.qaisar@seecs.edu.pk (S.B.Q.); husnain.saeed@seecs.edu.pk (H.S.); 2 Department of Computer Engineering, College of Computer and Information System, Umm Al-Qura University, Makkah 21514, Kingdom of Saudi Arabia; E-Mail: mfkhan@uqu.edu.sa; 3 Comsats Institute of Information Technology, Wah Cantt, 47040, Pakistan; E-Mail: muhammadnaeem@gmail.com; 4 Wincore Lab, Ryerson University, Toronto, ON M5B 2K3, Canada; E-Mail: alagan@ee.ryerson.ca

**Keywords:** cyber physical systems, condition monitoring, internet cloud, pipeline infrastructure, wireless sensor network

## Abstract

The synergy of computational and physical network components leading to the Internet of Things, Data and Services has been made feasible by the use of Cyber Physical Systems (CPSs). CPS engineering promises to impact system condition monitoring for a diverse range of fields from healthcare, manufacturing, and transportation to aerospace and warfare. CPS for environment monitoring applications completely transforms human-to-human, human-to-machine and machine-to-machine interactions with the use of Internet Cloud. A recent trend is to gain assistance from mergers between virtual networking and physical actuation to reliably perform all conventional and complex sensing and communication tasks. Oil and gas pipeline monitoring provides a novel example of the benefits of CPS, providing a reliable remote monitoring platform to leverage environment, strategic and economic benefits. In this paper, we evaluate the applications and technical requirements for seamlessly integrating CPS with sensor network plane from a reliability perspective and review the strategies for communicating information between remote monitoring sites and the widely deployed sensor nodes. Related challenges and issues in network architecture design and relevant protocols are also provided with classification. This is supported by a case study on implementing reliable monitoring of oil and gas pipeline installations. Network parameters like node-discovery, node-mobility, data security, link connectivity, data aggregation, information knowledge discovery and quality of service provisioning have been reviewed.

## 1. Introduction

Technological advancement in semiconductor design, material sciences and networking are driving the ubiquitous deployment of large scale wireless sensor and actuator networks [1]. Today, these technologies have merged to enable Wireless Sensor Networks (WSNs) that can provide low-cost, low power, multifunctional miniature devices with interfaces to connect with a multitude of sensors [2]. These devices can gather sensed information from the environment and communicate it in an untethered manner over a short distance that can be further routed by means of multi-hop to a central monitoring station. Applications of WSN have now found their way into the work place, home and environment enabling more user control and convenience. Significant research contributions have also made WSN communication more reliable for real time scenarios paving way for a well-established mix of software and hardware solutions in future applications [1].

**Figure 1:**
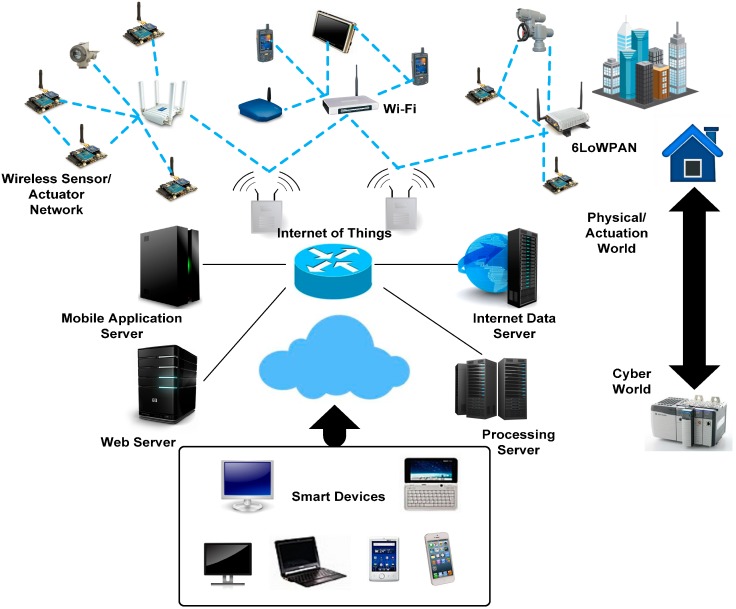
Network elements in CPS architecture.

With maturity in WSN protocols, researchers have started looking into extended functionality for interaction with other network systems using reliable and secure methods. Cyber Physical Systems (CPS) principles are thus finding their way into sensing applications as a platform to provide extended interactive functionality between real time and virtual environments. CPS provides an intuitive interconnection mechanism for human-to-human, human-to-machine and machine-to-machine interactions through the facilitation of seamless network connectivity and refined user control over actuation side [3]. By definition, CPS is meant to provide a virtual environment that incorporates an interacting network of system elements with physical inputs and outputs at both ends (Figure 1).

A WSN enabled with CPS can provide remote control over the network devices with scattered network elements instead of a standalone system [4,5]. Currently, only a vague interface portfolio exists to identify the overlapping areas of CPS and WSN where both can be seamlessly integrated. There is therefore a need to define clearly as to where the communication layers for WSN and CPS would connect and how to overcome the challenges in making both platforms compatible. With monitoring of oil and gas transfer installations using WSN and CPS as the main focus, we link real world challenges and industrial practices to provide a concise technical summary as to where cyber physical and sensor network mergers would stand in the future and directions to meet the requirements. With a concise summary of WSN and CPS platforms, oil and gas pipeline infrastructure health monitoring as well as environmental- and fluid condition-related reliability requirements are explained. The rest of the paper is arranged as follows: In Section 2, CPS based sensor network deployment is highlighted. In Section 3, Quality of Service (QoS) parameters in Cyber Physical Sensor Networks (CPSN) is explained. Section 4 deals with reliability and prediction design requirements for CPSN architecture. Section 5 is dedicated to integrating platforms, protocols and applications for CPSN, followed by a case study on oil and gas pipeline monitoring application in Section 6. Finally, Section 7 concludes the paper with a summary of CPSN for infrastructure monitoring.

WSNs have gained importance in recent years due to the proliferation of Micro-Electro-Mechanical Systems (MEMS) technology facilitating the fabrication of Smart Sensors. The miniature sized sensors with limited computing power can sense, gather information and measure several environment parameters and, based on the local decision, forward it to a base station. Though battery is the main source of power for these devices, recent techniques have started to focus on energy harvesting from natural resources like wind, solar energy and natural vibrations.

**Figure 2:**
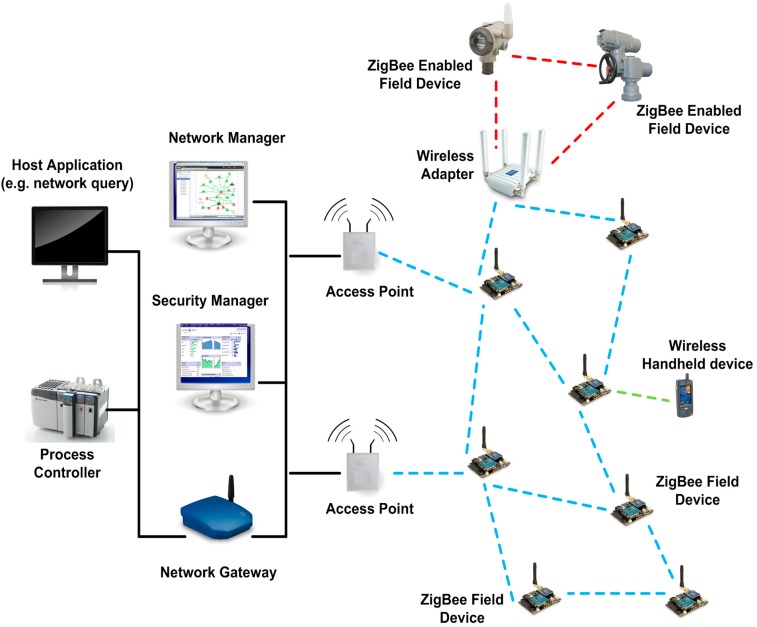
A typical wireless sensor network setup with central monitoring.

A typical WSN has little or no infrastructure and consists of numerous sensor nodes laid out over the monitoring area that gather data over time. For infrastructure based WSN, network entities like gateways, access points and network manager are required (Figure 2). Hence, a strict categorization for WSN deployment strategy is the structured and non-structured WSN [1]. Depending on the environment, WSNs can be classified into terrestrial WSN, underwater WSN, underground WSN, multi-media WSN and mobile WSN [2]. An unstructured WSN would consist of a dense collection of sensor nodes deployed in an *ad hoc* unattended manner. The unstructured WSNs are difficult to manage in terms of detecting connectivity failures [6]. The structured WSN nodes are deployed in a pre-defined manner at strategic locations, e.g., in a linear or hierarchical topology. Applications of WSN range from military tracking, environment surveillance, natural disaster relief, biomedical health monitoring and seismic sensing to hazardous environment exploration. A major classification of WSN applications lies in tracking and environmental monitoring scenarios [7].

Unlike traditional wireless networks and more intelligent radios that are capable of adopting communication parameters in run time, WSNs come with their own resource and design constraints [8,9]. Resource constraints include limited bandwidth, limited amount of energy, limited processing power, low storage capacity, and short communication range. Such constraints are in general not coupled with other wireless and cellular networks [10,11]; hence WSN require special deployment consideration from coverage perspective as well. Design constraints vary according to the application of WSN and the type of environment. Research in WSNs aims to meet these constraints by introducing new design concepts, improving existing protocols, developing new algorithms and testing applications in different scenarios. From a network management perspective and in terms of application requirements, it is important that the sensor nodes are capable of self-organization, *i.e.*, able to manage and control their actions in the network and their resource utilization. WSN standards that have been developed and widely adopted define the necessary protocols, functions and algorithms for sensor node interfaces to work without obstruction within the network realm. Some of the notable WSN standards include IEEE 82.15.4, ZigBee, WirelessHART, ISA100.11, IETF 6LowPAN, IEEE 802.15.3 and Wibree [1,7] (Table 1).

**Table 1:** Popular WSN platforms available for industrial and academic use [1,7].

Resource	Company/Major Distributor
***Sensor Nodes***	Argo Systems, WisMote, BTnode, IMote, KMote, TinyMote, EPIC Mote. EyesIFX, FlatMesh, Mica, Telos, Iris, NeoMote, Waspmote, RedBee, Ubimote, Shimmer, WizziMote, FireFly, Bitsym Bitsense
***Gateway Node***	AdvanticSys, Dwara, Shimmer Span, Stargate, FlatMesh,VEmesh, DigiMesh, Bitsym Bithaul
***Microcontrollers***	Texas Instruments, Atmel ATmega, ARM Cortex, Thumb Microcontroller Renesas, Marvell, PIC
***Transceivers***	Chipcon, ATmega, ZigBit, himmer,RFM
***Operating Systems***	Contiki, ERIKA Enterprise,Nano-RK, TinyOS, LiteOS, OpenTag, NanoQplus
***Programming Languages***	C, LabVIEW, nesC
***Software***	Arduino API, TinyDB, TOSSIM, NS-2, NS-3, OPNET, NetSim, LinuxMCE, QualNet
***Industry Standard Protocols***	ANT 6LoWPAN, DASH7, ONE-NET, ZigBee, Z-Wave, Wibree, WirelessHART, 802.15.4, MiWi

The possibility to develop a CPS enabled sensor network has arisen through the accelerated development of wireless technology and embedded computing with applications like micro sensing MEMS, inertial motion detection, bio-signal sensing, environment parameter sensing, location and vehicular movement detection [12]. While a single platform is being sought for defining common parameters, the major technical differences need to be emphasized. WSN has been designed and implemented mainly with the idea of communicating sensing related data with coordination over some limited geographical environment. CPS, on the other hand, utilizes a broader definition and dimension of sensing data over multiple networks (multiple WSNs) with a Cloud specific link to the Internet with the aim of providing flexible control and intelligence. Major technical requirements for CPS and WSN have been summarized in Table 2. CPSNs may encompass several WSNs; hence, the CPSN layer is technically able to support dynamic network formations, cross layer communications, larger geographic area coverage with looped actuation, mobility patterns and use of knowledge mining algorithms.

**Figure 3:**
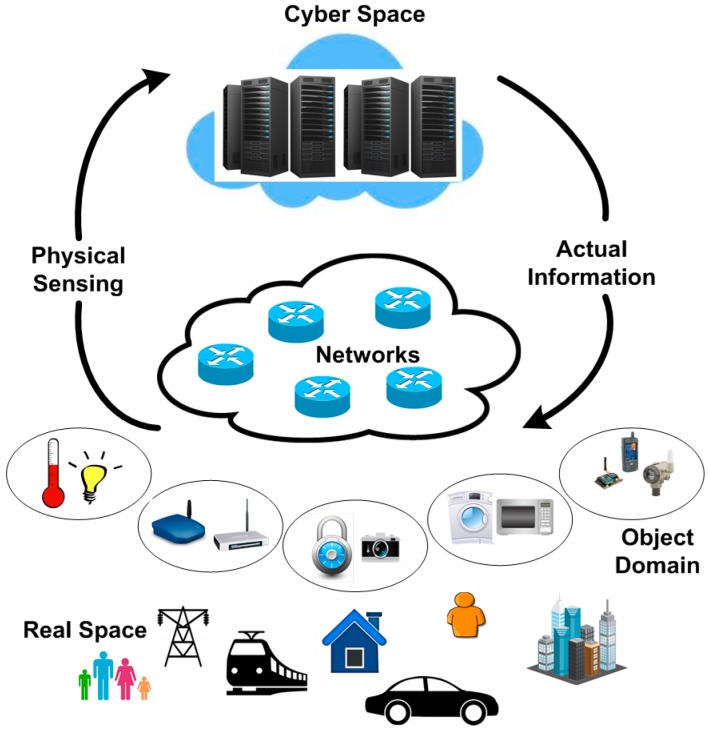
Cyber physical system applications with interconnecting boundary between cyber and object domain.

The CPS architecture resembles traditional embedded systems that aim to integrate abstract computations with physical processes. Contrary to traditional embedded systems, CPS provides an interconnected interaction with outputs and inputs that pertain to physical existence and are standalone devices (Figure 3). The main layers of CPS are the virtual layer and physical layer. For the physical layer, an intelligently deployed network of actuators and sensors collects information and actually controls the physical world. By converting the analog information into a digital format, the information is sent to a virtual layer input which serves as the decision-making setup. This information is further used to calculate abstract computations that feed into the real world actuation system to drive and control physical world outputs or objects.

**Table 2:** Technical requirements for cyber physical systems and wireless sensor network architectures.

Characteristic Aspect	Wireless Sensor Networks	Cyber Physical Systems
***Network Formation***	Field and application specific Less mobility support for infrastructure monitoring Supports joining and leaving of a node	Can encompass several overlapping networks from different applications Supports dynamic joining and leaving of a network
***Communication Pattern***	Data communicated to a central point Use of collective node effort to route data Mostly query and response interactions occur between nodes	Supports intra WSN communication Frequent cross layer interaction support for control over numerous actuators and QoS provisioning
***Power Management***	Power management is critical since system lifetime is associated with it Nodes are activated and put to sleep depending on the mission sensitivity and critical situations	CPS is supported by web cloud in most cases where power concerns may not be as critical due to abstraction of middleware network The monitoring station and actuation centre might need to be active most of the time
***Network Coverage***	Strict requirements to meet network coverage for specific environments Network needs to remain in connected state for long periods of time	Broader coverage and connectivity options that lie outside of WSN domain
***Node Mobility***	Both controlled and uncontrolled mobility of nodes can be used in a network	May include mobile and static node networks Data from nodes of hybrid interconnected networks collected in dynamic and random fashion.
***Knowledge Mining***	More focus is on collecting and aggregating sensed data	More elaborate information gathering and knowledge base population Intelligent decision making platforms dedicated to analyzing information.
***Quality of Service***	Quality of sensed data is important for WSN due to low data rate restrictions	QoS for CPS relates to a higher level cross layered approach Security and confidentiality are important service aspects

In contrast to CPS, WSN architecture focuses more on node design and inter-node communication and networking. Hence, WSN may be considered a subsystem of a largely deployed CPS architecture where the portion from gateway to remote monitoring station may be replaced with the Internet Cloud. Considering WSN as a standalone system, the basic sensor node is dedicated to measuring metrics from various environmental monitoring sensors related to physical, biomass and chemical parameters and converting them into digital information to be inferred by a remote monitoring facility. Form an application perspective, we can classify important resources in WSN node as the sensing unit, information processing element, transceiver device and power management module. Once WSN and CPS converge, communication and networking for CPSN needs to handle joining and departure of hundreds of nodes while providing scalability. Hence, for real-time data transportation in CPSN, re-configurability of nodes and manipulation of hardware during run-time needs to be taken into account [13,14]. Additional technical details of the networking parameters, architecture and related QoS measures in a CPSN are discussed in subsequent sections.

## 2. Cyber Physical Senor Network Deployment

A typical approach to highlight Cyber Physical Sensor Network (CPSN) deployment issues is to discuss the system from the bottom-up, *i.e.*, from the physical sensing plane towards the monitoring site over the Cloud. The available WSN platforms can replace the sensing domain of CPSN without major changes; however, the gateway interface must be able to manipulate the commands from and to the Cloud seamlessly, considering the sensor nodes to be intelligent enough to manipulate them. Considering WSN communication protocols, the IEEE 802.15.4 PHY and MAC has been the most widely used data-link standard in WSN deployments while the ZigBee protocol which is an extension of IEEE 802.15.4 further provides support for distributed addressing and use of tree topologies [1,7]. The deployment of CPS enabled gateways for WSN can be further leveraged by use of internet-ready sensor gateway devices, where a recent trend is to even provide open source IPv6 compatible software extensions with the devices [15].

For extensive data gathering requirements in the CPSN physical domain, peer-to-peer communication may have higher overheads; hence, a low packet exchange mechanism may be used. The Convergecast communication mechanism, where sink nodes are required to frequently gather sensing data from a set of nodes, might be considered a better mechanism for CPSN considering an intelligent gateway residing at the CPS and WSN boundary. Communication in CPSN physical monitoring environments can be broadly classified as: (1) cluster-based; (2) schedule-based and (3) correlation-based communication methods. The cluster-based solution works on a partitioning mechanism where sensor nodes are divided into groups with a leading data collection and aggregation node. The schedule-based solution arranges communication timings between nodes with the goal of achieving overall low latency and energy consumption. Finally, the correlation-based mechanism exploits relationships in spatial and temporal domains between different nodes to reduce redundant data transmissions.

Data dissemination in the sensor network domain of CPSN is an important aspect. To enable consistent communication reliability in CPSN sensing plane throughout the monitoring period, a query-and-reply method between nodes is used frequently that may become difficult to handle in complex topologies with thousands of nodes. This step of data dissemination with communication reliability can thus be carried out in more intelligent ways all of which can be broadly categorized under query-and-response. We mainly classify query-and-response based data dissemination into three categories: (1) directed diffusion; (2) distributed indexing and (3) multi-resolution methods [1,2]. *Directed diffusion* is a data centric information dissemination method. Data generated by sensor nodes is given in the form of attribute-value pairs. A node requests data by sending interest messages for named data. Data matching the interest is then drawn and routed towards that node. Intermediate nodes that fall in the data path can cache, or transform data, and may even direct interests based on previously cached data information. *Distributed indexing* on the other hand uses a distributed addressing method to gather information. For more complex topologies, hybrid query resolution approaches may be adopted that are a mixture of diffusion and address indexing methods.

**Figure 4:**
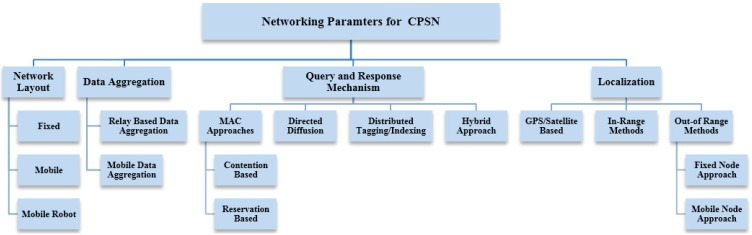
Network communication parameters for cyber physical sensor network architecture.

Avoiding disconnections and providing extensive coverage is another essential design parameter that affects the system performance. In a simple CPSN sensing node with plane layouts and theoretical design, sensors may be assumed to have fixed communication and sensing ranges initially. In a simplistic manner, the coverage problem for CPSN can be formulated as a decision problem where, given a number of sensors to be deployed in the field, the main target is to determine if the area is sufficiently *n*-covered, *i.e.*, every point in the monitoring field is covered by at-least *n* sensors, *n* being an integer. Sensing plane coverage solutions for CPSN can be categorized into opportunistic sensor node selection mechanisms or covering-set methods that utilize graph theory. Finally, sensor node localization can be done using Global Positioning System (GPS) related methods for outdoor deployments or through trilateration, proximity and other out-of-range methods including fixed anchor nodes (Figure 4).

**Figure 5:**
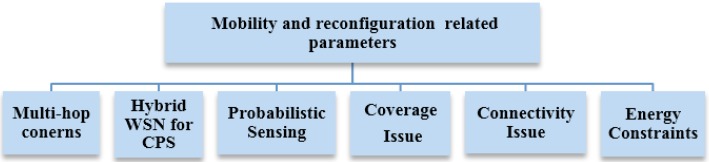
Wireless sensor network parameters and issues related to mobility and reconfiguration in a cyber-physical environment.

One important network parameter that concerns CPSN deployment is the node mobility that needs to be properly accounted for in the networking information for dynamic link perspectives and mobile robots deployed over the monitoring area. Mobility allows network capability to be improved in many ways, e.g., the use of automatic node deployment, flexibly adjusting the topology and rapid detection of events. Overall, such mobility related solutions for CPSN can be classified into two types where the first one would be to try relocating sensor nodes to improve network coverage and connectivity while the other solution would be to try addressing the path planning issues for data relaying nodes that would ultimately allow extended network lifetime [16]. Highlights of the mobility and reconfiguration related parameters, issues and research dimensions for CPSN are listed in Figure 5.

## 3. Quality of Service Reliability in Cyber Physical Sensor Network

QoS provisioning becomes a much complex task when the sensing plane of CPSN needs to address thousands of sensor nodes placed in various topologies [17]. These challenges need to be addressed on both sides of the gateway, *i.e.*, the Internet Cloud side and the sensing plane. QoS design requirements for implementing a planned CPSN would include: (1) Service Oriented Architecture (SOA); (2) QoS aware communication and networking; (3) intelligent resource management; and (4) QoS aware power management at various communication levels (Open Systems Interconnection “OSI” layers) [17]. SOA plays an important role in reducing the complexity of the infrastructure by decomposing CPS functions into smaller distinguishable units each viewed as a separate service [18]. This allows rapid, efficient and scalable development of a CPS application through reusable service units. Application level QoS, however, needs to be defined according to the service for intelligent cross layered communication. With the future trends in user applications [19], CPSN environments would require dynamic system settings for unpredictable environments [20]. Self-management policies would be needed so that allocated resources like CPU time, bandwidth memory, energy profiles can be controlled intelligently in a high level QoS constrained system setup [21,22]. A major concern with QoS provisioning also pertains to the minimization of energy consumption for major network elements. In this regard, Cloud Computing provides a possible solution wherein major enabling technologies for such a setup are Virtualization and Ubiquitous connectivity. Virtualization related technologies like Software Defined Networking (SDN) and virtual operating systems and connections allow provision of dynamically changing and altering resources based upon service isolation, thus enabling scaling and managing of resources in a more controlled way.

Since the resources of the cloud connecting the sensing platform are dynamically manipulated, the cloud itself would provision the typical three types of services to the sensor platform on one side and the remote user on the other side. These services include SaaS (Software as a Service), PaaS (Platform as a Service) and IaaS (Infrastructure as a Service). SaaS provides services to remote users on a demand basis. PaaS provides a development environment that is encapsulated and offered to users as a service wherein higher level applications can work over it. Finally, IaaS is responsible for provisioning computing capabilities and basic storage as standardized services for both sides of the network. Once the sensing platforms run under a cloud, several differentiations can be made according to the sensing application in hand. Since integrating WSNs with the cloud makes it easy to share and analyze real time sensor, further advantage can be taken by provisioning sensor data or sensor event as a service over the internet; hence, the terms Sensing as a Service (SaaS) and Sensor Event as a Service (SEaaS) are sometimes used. Some typical examples of protocols that could be implemented at different OSI layers for enabling CPSNs are mentioned in Table 3.

**Table 3:** Example implementation layers and protocols for a cyber-physical sensor network environment.

Implementation Layers	Protocol	Scope
Application	HTTP, COAP	End to End
Transport	UDP, TCP	End to End
Network	IP	End to End
Routing	RPL	Per Hop
PAN	6LowPAN	None
Data Link	IEEE 802.15.4	Per Hop

**Figure 6:**
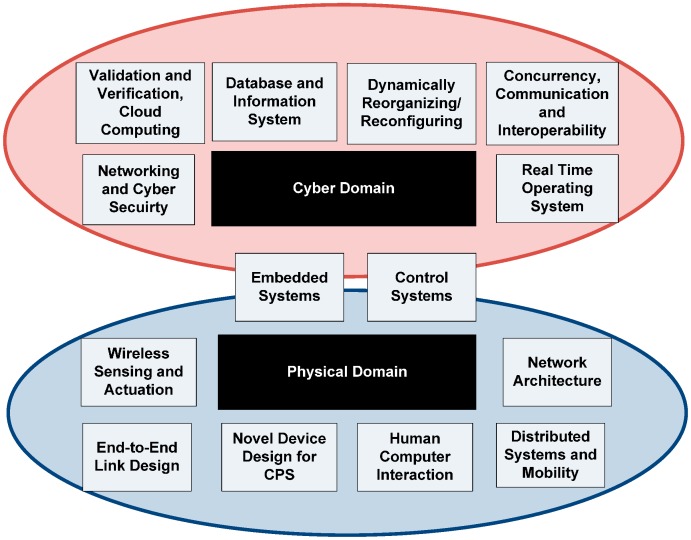
A division of cyber-physical sensor network requirements.

Prediction of accurate output decisions and reliability of sensing information are considered critical for CPSN systems. There is therefore a need to define strictly the network requirement factors in terms of cyber and physical domains (Figure 6). These factors also form the QoS basis for achieving a real time intelligent system for high stress and constrained environments like mining, healthcare and warfare. QoS factors like seamless data flows through the cloud and timely delivery at the monitoring station are considered critical for cyber systems. This becomes more challenging when CPS is integrated with other technologies like semantic agents and hybrid system states in the Cloud. Deployment of CPSN architectural parts require placement of sensing and actuator devices at strategically critical points with intelligent algorithms for node localization and geo-location detection. The Medium Access Control (MAC) at the sensing side should consider that the negotiation between neighboring data collection devices and sensors must conserve resources like bandwidth, number of channels, buffer storage and transmission energy [23].

Software, hardware, middleware and operating systems are required that provide reliability beyond existing technologies. The software and hardware must be highly dependable, certifiable, configurable and, where required, be able to fully integrate with complex systems. A complex CPSN system must possess reliability that is currently lacking in many deployments. Though overdesign is currently the safest path for system deployment, this approach becomes intractable for complex designs and systems where interoperability is required. Evidence based methods are needed for reasoning inference about system reliability. New methods, algorithms, models and tools are needed that can incorporate verification and validation of software and systems at the control stage.

A major focus must be on systems which are verifiably robust in order to keep operating under situations and environments with uncertainty combined with potentially rapidly changing objectives. CPSNs built for optimization, control and scheduling will have to interoperate efficiently in real time. Hence, further study is required to explore the organizing principles of such interactions and appropriate abstractions that can support services with significantly shortened design cycles.

Before CPSN implementation, it is important to develop representative models of all agents including electrical, mechanical and computational components as well as important environmental factors which influence the dynamics of the system. With the availability of a high level of computational power, several tools have also emerged for direct modeling of dynamic systems. Issues that dominate the reliability and prediction design requirements include: (i) verification of system wide safety/reliability properties; (ii) predictable interaction between cyber (communication/computation) and electromechanical (physical) components; (iii) computational tractability including algorithms that can scale; (iv) health, state and information management *via* communication over wireless and wired links.

## 4. Enabling Applications and Platforms for Cyber Physical Sensor Network

For monitoring remote sensor applications over the IP framework, cloud computing can provide a middleware cost effective solution to CPSN that provides a rich interactive communication platform. Since network communication costs a lot of bandwidth overhead for linking Virtual Machines (VMs) in data intensive environments, a decentralized approach, where migration of VM services is provided with monitoring of traffic fingerprints can relieve the wasted overhead. Also, in particular cases, faults can occur in the middle of a query from distributed databases. This can be fixed by dividing queries into sub-queries and mapping them in an intelligent way such that the results return on different nodes.

A global middleware concept can be a convenient way to provide flexibility integration and discovery of sensor networks and sensor data. Such a middleware would be required to provide fast deployment of testbeds with distributed querying, filtering and aggregation of sensor data with support for dynamic adaptation of the system configuration parameters. This would be linked to the use of virtual sensor abstraction that can enable users to declaratively specify deployment descriptions in an open standard human readable language like XML. Such an approach becomes more powerful for remote monitoring once there is a possibility of integration of sensor network data through querying language like SQL over local and remotely available sensor network resources.

Sensor Modeling Language (SML) can be used to represent any of the physical sensor’s metadata like their accuracy, type, physical location and similar measures. In addition to SML, XML encoding will be used for the measurement and description of the physical sensor specifications. The use of XML language allows encoding the sensors in a way that the implementation is available across several hardware platforms and operating systems through simple translation or use of a wrapper. A map for translating between physical and virtual sensor parameters can be used to translate commands.

The concept of metadata in Sensor-Cloud perspective holds the importance of publishing the type and location of the sensor at the time of data generation. In CPSN, location for different data generating and terminating points would serve as a first class knowledge for many relevant applications [24]. Compared to outdoor location detection through GPS and similar approaches, indoor location estimation or localization proves more challenging. Typical systems proposed in this context such as the Bat and Cricket [25] relate to smaller scale environments, while to cope with newer demands of extended scaled up systems, pattern matching of data can be applied as a useful solution. Locating the event on the relevant sensor node when requested by a remote application is an important issue. Several methods have been proposed in the literature to account this issue. One method is to locate the physical sensors with data faults by assuming a mismatch between the sensor data rank and the distance rank.

**Figure 7:**
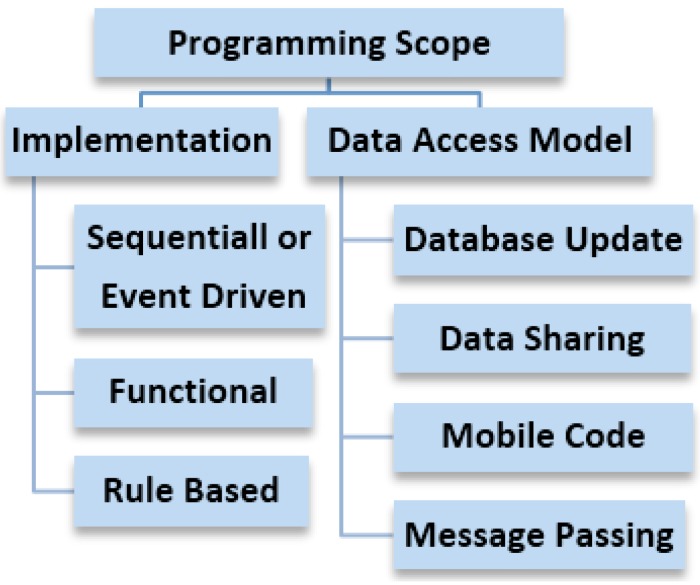
Data processing related programming scope for remote sensor network monitoring.

**Figure 8:**
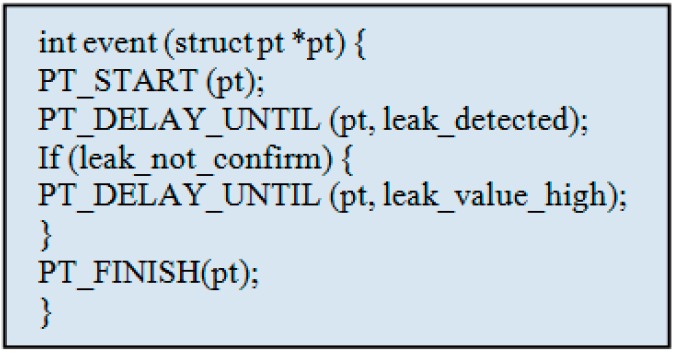
Event driven code design abstraction for data sensing model.

The command translating agent at the gateway side linked to the Cloud would directly depend upon the CPSN programming scope for implementation of remote sensor network monitoring using cyber physical systems [26]. Major aspects in the middleware programming scope are highlighted in Figure 7 where the code implementation can be done in a number of ways depending upon the sensor data and processing requirements. Typical sensor data processing implementations include sequential, event driven, functional and rule based filtering. An example of typical programming instance to be run on data sensing model is given in Figure 8 that uses an event driven approach to detect a sensor value that is categorized as a leak. The data access model for connecting the data base can be implemented using several combinations of steps like message parsing and use of mobile code for timely data retrieval (Figure 7).

CPSN is actually a bridge to link the cyber world with communication, intelligence and information components with the physical world counterpart providing sensing and actuation capabilities [27]. The CPSN platform may be broadly classified as an integration of an intelligent control design system with a mobile or static sensor or actuator system. When considering standalone individual sensor networks, issues like network formation, security, mobility and power management remain almost the same in a broader perspective. However, major technical differences for the CPSN approach include the use of heterogeneous information flow, multi-dimensional sensor cooperation and a high level of intelligence and algorithms informing the actuation and decision framework.

**Figure 9:**
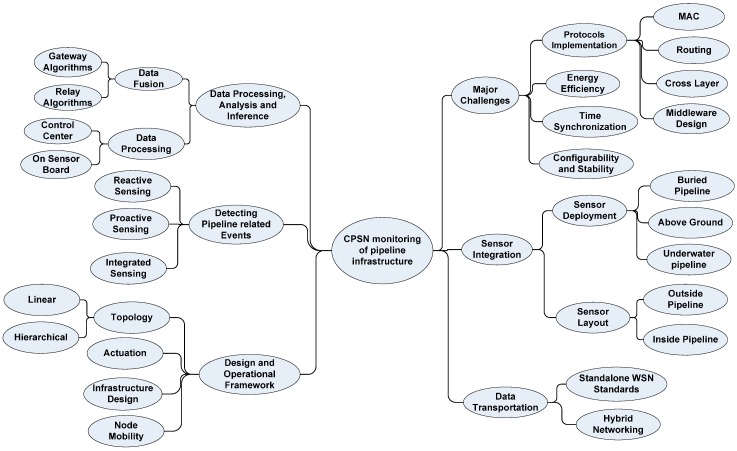
Summary of wireless sensor network based cyber physical monitoring of pipeline infrastructure.

From the service applications’ viewpoint, the cyber system itself has a wide range of useful features that can be used to provide elevated services to users with numerous implementation opportunities. For example, a complete CPSN can be used to assist in management of greenhouse sensing information at an extremely large geographical distance. More complex systems could include multiple sensors and actuators that can be used for applications such as environment-related climate control settings with humidity, heating, carbon dioxide generation, fertilizing and watering system features [28] (Table 4). A summary of the enabling applications and platforms required for CPSN is given in Figure 9.

**Table 4:** Applications highlights of sensing plane in cyber physical sensor network.

WSN in a CPS Environment: Applications and Platforms		
**Applications**	Health Care	Physical Rehabilitation
		Telemedicine
		Elder Assistance
	Security	Transit Tracking
		Emergency Navigation
	Social Networking	Personal Interaction
	Gaming	Grouping, Interaction
	Monitoring	Traffic Lights
		Parking
		Route and Transit Planning
**Sensor Types**	Pulse	ECG, EMG
	Movement	Accelerometer, Tilt Sensors
	Imaging	Video Camera
	Thermal Visibility	Light, Temperature Sensor
	Location	GPS, Compass, Magnetometer
	Commodity	Gas and Liquid Monitor
	Frequency	Ultrasonic’s
**Communication Platforms**	Long Haul	GPRS, LTE, 3G,
	Limited	Body Sensor Network

Important elements for CPSN based pipeline infrastructure monitoring require intelligent sensor integration according to the pipeline layout, correct sensor event detection algorithms, data fusion and inference techniques, data routing across the network and cloud interfaces. Pipelines can be underground as well as above ground; hence, sensors calibration and positioning would be affected by atmospheric phenomenon like winds, humidity and day period. Quite recently, the use of acoustic sensors for underwater environment monitoring has become popular where several research groups are actively deploying them in experimental testbeds like coral reef monitoring and underwater pipelines [29,30,31]. Once the sensors are in place, the sensing algorithms play an important role in timely detection of sensor events using reactive, proactive or mixed algorithm trigger mechanisms.

Once the events are detected from multiple sensors, diverse information on a node can be fused at sensor node for compressing data following the sensor network data rates being typically low. The data can also be fused at relay or gateway nodes in case the CPSN encompasses several sub sensor networks. In addition to data fusion, data processing codes can be found in a distributed manner where one code instance runs on the sensor nodes and another runs on the remote control station. Overall, design of a CPS based wireless sensor network would require topology controlled infrastructure design, actuation mechanisms, an intelligent middleware lying in the Cloud and a data routing mechanism from the sensor node towards the remote monitoring station, as well as feedback from the controller to the actuation platform.

## 5. A Case Study on Reliable Pipeline Monitoring Using Cyber Physical Sensor Networks

Oil and gas pipeline monitoring provides a novel example where WSN can provide remote monitoring while CPS integration can be leveraged to apply real time information and analytics of the underlying framework [32]. With a number of possible pipeline deployment techniques available, all efforts unswervingly reflect the characteristics of the medium needed for transportation that depends upon the environmental, strategic and economic conditions. For intelligently providing flexible and reliable CPSN-based monitoring, factors like sensor layout, data transmission methods, sensor node power concerns, data processing, analysis and inference points, operational design and framework in addition to network topology, infrastructure and sensing related technologies are the focus of this section.

The oil and gas distribution pipeline deployment methods can be widely characterized into underwater, above ground and buried pipelines. Since leakages could be detrimental to the surroundings, integral pipeline monitoring is essential and needs to be reliable and in real time. The main sensing techniques can be categorized into reactive ones that detect the presence of leaks only once they occur while the proactive methods monitor the condition of pipelines gradually over time to prevent leakage occurrence [33].

### 5.1. Sensor Layout

The type of sensors, their placement and their usage in the monitoring environment form the basis for CPSN monitoring systems. Sensors placement for pipeline monitoring can be classified as outside placement and inside placement that may use invasive or pervasive techniques depending on the scenarios given [34]. Monitoring changes in pipelines with visual inspection is a handy technique for monitoring events that occur above ground. Vision sensors allow distinguishing differences or changes in the area around the pipeline. With such sensors, small changes in the physical nature of the pipelines, temperature difference or the event of any fluid or gas leakage can be easily determined. Ground-penetrating radar technique is another widely used method to accurately monitor changes and collect evidence of the existence of any occurrences at the ground level without digging. With the help of acoustic transducers, small fluid or gas leaks are easily identified as they produce frequency oscillations [35,36]. The coverage range of these transducers is very small; hence, a number of transducers are required to cover the desired underground pipeline requiring extensive operational and maintenance efforts. To reduce such efforts, acoustic transducers are only deployed on the pumping stations or near checkpoints. With the mass balancing method [36], the difference in flow of entering and leaving fluid can be monitored though it is not an efficient method for locating major leakages in underground pipelines.

### 5.2. Sensing Techniques for Pipeline Monitoring

**Figure 10:**
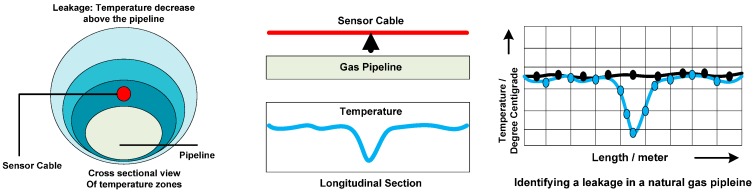
Optical fiber-based leakage detection mechanism.

One of the ways to detect leaks is by comparing the change in temperature profiles of the immediate surroundings of the pipeline due to the Joule Thompson Effect [37]. For compressed cases such as Liquefied Natural Gas (LNG), the difference is up to −190 °C, but in case of underwater pipelines, there is only a subtle change in temperature. Temperature sensors for this purpose should be selected keeping the temperature gradient in mind. Distributed fiber optic sensors [38] provide a temperature signature and pinpoint the leak location. Optical fiber sensors need to be in close contact with the pipe so as to come in contact with a leaked fluid (Figure 10).

Figure 10 illustrates an optical fiber-based leakage detection mechanism for gas pipelines using a distributed acoustic sensing technique that measure the backscatter that results due to disturbance caused in the fiber. The normal temperature profile and the temperature profile sensed from the fiber sensor in the presence of a leak are also illustrated. The fiber sensor is placed above the pipeline for gases and below the pipeline for liquids [13]. This sensor provides an effective solution for pipeline monitoring because of its low cost and easy installation. One attractive feature of optical fiber sensing is the fact that the optical fibers have already been installed and used for communication purposes along the pipelines [38]. Several techniques have been proposed for under-ground sensing of pipelines. Sensing soil properties can be useful to find abnormalities in soil [14]. Sensors can detect temperature variation in the soil due to leakage of hot liquids [36,39]. Hydrocarbon vapor sensors can be used to detect the leakage of pipelines transporting liquefied natural gas [39]. Soil dielectric property sensors can be used to detect the leakage of crude oil pipelines [40,41]. Acoustic sensors can be used to detect leaks in municipal water pipes, sewages, and oil and gas pipelines, respectively [29,30,31].

Since corrosion in pipelines makes the inner pipe surface vulnerable to leakages and any external or internal strike (like solid particles hitting the surface with high velocities) can result in leakages, it is also necessary to monitor pipes flowing fluid for the presence of solid particles. Using acoustic sensors to detect the presence of solid impurities such as sand in oil, the sensors are placed non-intrusively near the bends of the pipeline [42]. The solid particles collide with the surface of the pipeline at the bends which generate high frequency waves that are detected by the sensors. Mobile sensor robots move inside the pipeline to monitor pipeline conditions and to provide accurate measurements and readings about the defects appearing inside the pipeline at regular intervals. Industries are therefore interested in developing robots with ingenious designs so the whole pipeline can be scrutinized [43].

A recently developed Magnetostrictive Sensor (MsS) inspection system [44] is an intrusive sensor which detects corrosion in the pipelines. Research work in [45] describes Carnegie Mellon’s robot Explorer II which is designed to be used in pipelines of different diameters as it has adjustable diameter and contains a number of inexpensive piezoelectric sensors to pinpoint leaks. In the research work described in [46], four different topologies of Multifunctional Robot for In-Pipe Inspection (MRINSPECT) are investigated. The research contribution in [47] identifies construction equipment to be one of the major causes of breakages of pipelines and proposes efficient acoustic sensing with noise cancellation that can detect the presence of such equipment and generate an alarm at the base station. PipeSense [48] has provided an alternate sensing method to acoustic sensing by incorporating induction based *ad hoc* RFID wireless sensor networks for water pipelines. Meanwhile, work described in [48,49] uses pressure signals to measure the events like leakages and bursts by monitoring the signal states using stochastic HMM processes. SWATS [50], a system for monitoring steam flood and water flood pipelines, makes use of all common measurements such as pressure, flow and temperature. Work in [51] describes a thermal video technology for leak detection. Thermal cameras are used to exploit temperature differentials which provide greater accuracy than methods that rely on color and size characteristics.

### 5.3. In-Pipeline Leak Monitoring

For problems created inside the pipeline, several in-pipeline monitoring applications have been stated in the literature. Such problems resulting in leakages may be caused by sudden changes in pressure, corrosion, cracks, bad workmanship, defects in pipes or lack of maintenance [52]. Since the detection system comes closer to the location where the leakage is inside the pipeline, the in-pipeline leakage detection methods are considered more accurate and less sensitive to external noise. The Smartball [53] as a mobile sensor device can be used to detect and locate small leaks inside pipelines that are larger than 6 inches in diameter. The sensor device is developed in the form of a free swimming device consisting of a porous foam ball that envelopes an aluminium sphere containing the sensitive acoustic instrumentation. Another in-pipeline monitoring device, Sahara [54], is able to estimate and locate the leak in large diameter water pipelines. The system travels by the flow of the water and, in case of a leak, the exact location is marked on the surface by an operator that follows the machine movement. Both Sahara and Smartball are passive since they cannot be actuated and cannot actually maneuver inside the pipeline. Several crawlers have also been reported that utilize wheeled platforms, cameras, and a mechanism for control and communication, e.g., the *MRINSPECT* [55].

For in-pipeline leak detection involving gas, the Explorer [56] has been used which is a long range leak inspection robot controlled by a human operator *via* wireless RF signals. The explorer constantly looks into an installed camera to search for leaks that are useful for offline inspection. For oil pipelines, nondestructive inspection is the most successful technique. Magnetic flux leakage based detectors and ultrasounds are used quite commonly to search for pipe defects. Major fault with such an approach is the dependency on pipe material and high power usage. These are also not suitable for long range mission where maneuvering capabilities are difficult due to large pipeline size. PipeGuard is able to detect leaks in a more reliable and autonomous fashion as compared to passive approaches [52]. The PipeGuard system is inserted into the network through special insertion points and the system reports leakages wirelessly through relay stations. Leakages are detected by identifying pressure gradient in the leak’s vicinity.

### 5.4. Data Transmission and Delivery

After the parameters from sensors are successfully measured and recorded, a mechanism to transmit the data to the base station is required. Reliable and secure transmission of data is significantly important. For this purpose, various network architecture and topologies have been proposed [57,58]. Factors such as real time sensing node design, pipeline and network infrastructure, connectivity of nodes to base station and battery life or duty cycle directly affect data transmission [59].

Several wireless networking standards such as Bluetooth, ZigBee, and DASH7 are frequently used in integration for sensor network implementation. For pipelines situated in remote areas, it is desirable to make use of long range networks like GSM and GPRS to transmit data collected on the backhaul network. Wireless HART (WiHART) [36] technology has been used as the first open wireless standard for industrial process control in sensor networks. For infrastructure monitoring of linear and hierarchical designs, different wireless standards of transmission may be employed at different hierarchy levels.

The choice of the transmission standard will depend on the cost and the desired range. Wireless signals get severely attenuated in the underground and underwater transmission resulting in unreliability. The effect of extreme path loss, reflection/refraction, multi-path fading, reduced propagation velocity, and noise on the propagation of electromagnetic waves in underground networks has been described in [39]. Several alternatives to underground problems have been provided in [60,61] that make use of electro-magnetic induction for transmitting signals. Underground communication may also be improved by activating the soil property sensors only when a leak is suspected and by wiring the processing hubs to the coils that prevent transmission losses. Acoustics can also be used for communication wherein the details on the effect of path loss, noise, multi-path fading, doppler spread, and long and variable propagation delay on the acoustic signals used for pipelines underwater can be found in [62].

Before sending the sensor readings over to base station, the gateway node needs to compress the information in a meaningful form to transfer maximum information on limited bandwidth [63]. This includes extracting several features from the bulk of data such as mean and variance. The techniques used for data aggregation discussed in [18,64] include Fuzzy ART, Maximum Likelihood Estimator (MLE) and Moving Average Filter (MAF). Fuzzy ART implements a neural network module that classifies the available information into groups and assigns probabilities to each group in order to make decisions for the incoming data based on those probabilities [64]. In case of MLE, the data observed from all sensors is used to create a likelihood function. The function is then maximized over all possible values of mean and standard deviation. MAF method computes average of the inputs to provide an output value that is useful in noisy channels where input values differ a lot from the mean of averaged out scores [65].

After the fused and compressed meaningful data reaches the base station, an algorithm is run on it to make an inference about the condition of the pipeline and the carried fluid or gas. The algorithms differ based on the type of sensing technique applied and the parameter that is under consideration. Haar Wavelet transform can be used to detect pressure pulses that localize leaks in a pipeline with reduced complexity [66]. The acoustic/vibration signals are cross correlated to detect leakages. The challenge in this technique is to synchronize the signals in time to make an adequate deduction. The delay of receiving the HF noise caused by the leaks at different nodes can be calculated by their physical distance and speed of the acoustic signals traveling across the pipe.

### 5.5. Architecture for Remote Pipeline Monitoring

**Figure 11:**
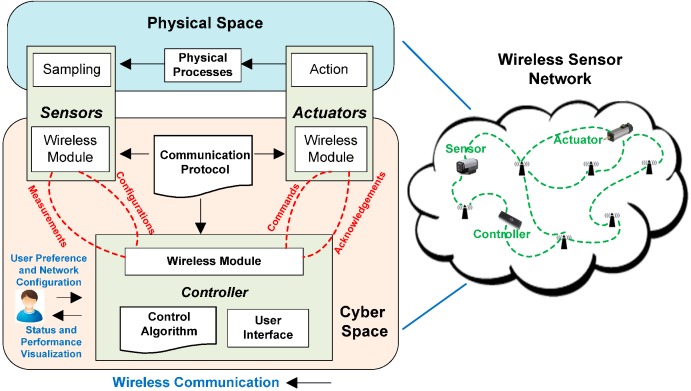
Interacting elements of cyber physical sensor network for infrastructure monitoring.

The structured WSN deployment on pipeline simplifies the routing protocols and increases efficiency and cost-effectiveness of the network. A hierarchical structure for pipeline monitoring based on sensor networks has been described in [40] wherein the functionalities are distributed among the nodes. The nodes at the lowest level are the sensing nodes that sense the parameters from the environment [67]. Relay nodes closer to sensing nodes collect sensed data and pass it on to data dissemination nodes which finally transmit the data over long haul communication links to the control center. The advantage of such a topology is that it adds redundancy to the whole architecture while reducing the range of each node that ultimately induces low energy consumption [68]. The functionalities of nodes on each level can be made distinctively diverse so as to make the data collecting system more intelligent (Figure 11).

Data relay nodes require larger memory compared to the sensing nodes while the data dissemination nodes require transmitting at longer distances. The hierarchy based architecture helps adding extra features only in the places where required. Work described in [40] also emphasizes having more than one node in the transmission range of any arbitrary node so as to add redundancy and prevent disconnection for node failures. Moreover, it has been noted in [41] that the nodes’ power consumption increases depending on the nodes proximity to the main node on a pipeline. Nodes’ power consumption doubles for every next hop [69]. If the gateway node is placed on the right most part, the left most node utilizes minimum power and the one nearest to the main gateway node utilizes the maximum due to data aggregation over several hops.

In order to prevent excess power consumption and maximize the network lifetime, work described in [41] proposes equal-power placement schemes to improve the WSNs lifetime by up to 29% with a properly selected number of sensor nodes and adjusting the distance corresponding to transmission power levels. WSN require the capability to reorganize when the new nodes are added to a network, and it should be able to heal when nodes die out due to any foreseen reason (battery damage *etc.*). Routing protocols must be intelligent enough to cope with such situations. For a hierarchical WSN with tree routing algorithm including self-organizing and self-healing network capability, a transport level fragmentation of large sized packets to prevent loss of information is required [66].

Oil and gas sector of different countries in the world has excelled in the technology and using CPSN based monitoring system to gather up-to-date information of its different processing stations. Besides real-time monitoring of various stations, a number of countries are also using wireless sensor nodes to collaborate accurate reading from different operational points. We conducted field visits of oil refineries and gas industries in the Middle East, who are using CPSN based monitoring systems for many years. These surveys were conducted in the vicinity of Saudi Aramco refineries in KSA (Yanboh Refinery run by Saudi Aramco Luberef), Kohinoor Mills gas distribution plants, ExxonMobil and Pak-Arab refinery. Information reported in this paper is gathered by field visits and provided by trained and experienced technical staff of the respective industries. Insights gained through industrial visits show that important WSN features such as mobility of the system and nodes, knowledge of node attachment locality in the operational plant, selection of network topology, scale of the network, availability of router nodes and battery lifetime, physical separation among nodes, and reliability and flexibility of nodes, and their operations define the overall system reliability.

Practically, refineries and gas distribution fields do not follow any specific strategy in sensing nodes’ deployment. Hence, no specific predefined network topology exists and a random deployment is followed across the length of the pipeline. Also, in the oil industry, the position of the wireless nodes is not changed very often. Hence, sensing nodes are deployed only once and replaced from their locations only if they malfunction or get critically damaged. Integrated sensors such as fluid level indicators, pressure, temperature and fire detection sensors are deployed according to specific industry requirements for certain places (Table 5).

**Table 5:** Sensor devices used in industry for event detection.

Sensor Used	Fluid Inside Pipeline	Sensor Placement	Events Detected by Sensor	Physical Phenomenon
Acoustic sensor [29]	Any	Outside the pipe	High frequency noise	Leakage
Acoustic sensors [29]	Any	Outside the pipeline near the bends	High frequency noise	Presence of solid particles in the fluids
pH sensor [30]	Water	Inside the pipeline-in contact with the fluid	pH variation	Contamination
Optical Fiber sensor [38]	Gas (Natural Gas)	Above the pipeline	All Major	Leakage
Optical Fiber sensor [38]	Liquid (Water/Oil)	Beneath the pipeline	All Major	Leakage
Hydrocarbon vapour sensor [39]	Oil	Outside the pipeline for underground pipelines	Change in the concentration of hydrocarbon vapor	Leakage
Magnetostrictive Sensor MsS [44]	Any	Inside the pipe	Strain upon the piezo-electric sensors	Rust/Blockage
Pressure Sensors [66]	Any	In contact with fluid	Fluid Pressure	Leakage

Oil and gas pipeline monitoring systems do not use wireless sensing nodes in bulk; instead, selected nodes are installed on predefined locations. Fluid level indicators, pressure and temperature based wireless sensor nodes are deployed in production plants that are usually fixed. In the oil industry, node mobility is not required because sensing nodes are placed at a fixed location for calibration of various real-time changes in the condition and state of the product.

**Figure 12:**
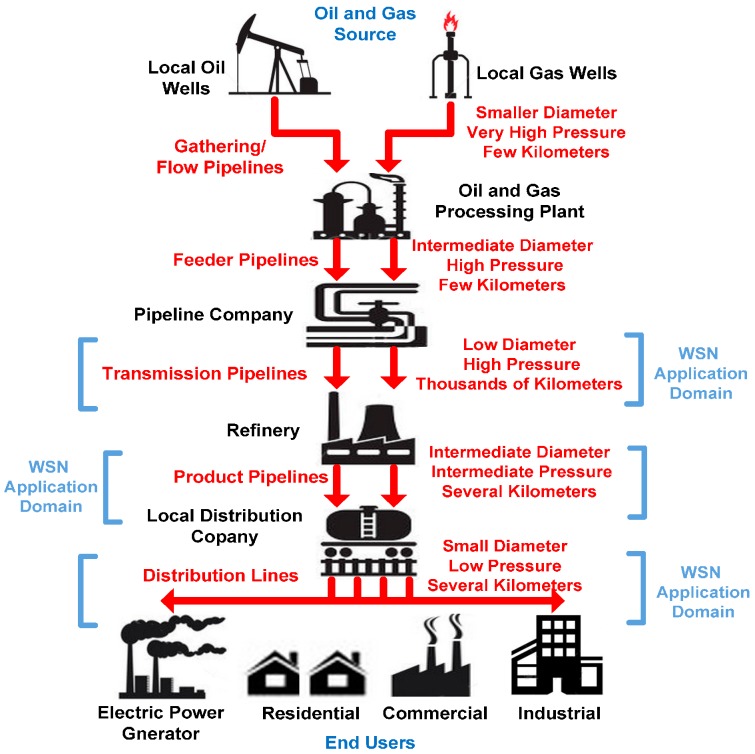
Sample pipeline configuration levels for oil and gas transfer.

In pipeline monitoring systems, the sensor plane is designed in such a way that each node directly communicates to the base station. For industrial environments, wireless sensor gateways are installed which take care of transporting the data to the base station. Every wireless sensor gateway is responsible for collecting data from its specified units. From surveys, it is concluded that it is preferable that sensor gateways are installed in an operation room. The distances between the wireless sensor gateways and the sensor nodes are kept at around 100 m. The deployment would also depend on the specific part being monitored in the complete fluid delivery system. Generally, the layout would form linear or hierarchical topology since the coverage area would vary according to the type of industrial pipeline layout depicted in Figure 12. By communicating with each other, they actually provide redundancy to the network architecture.

A major factor while installing sensing nodes in an industrial environment is the location to be monitored with the sensing device rather than deploying sensing nodes as per sleek network topology. Network topology does play an important role in data transportation and processing but somehow, in sensitive and critical networks, the location is more important than the design of the network. However, as a general rule, in oil and gas pipeline monitoring systems, nodes are deployed as per requirements and according to the need of the operation unit. The maximum number of hops observed between two designated nodes was one and the distance between each neighboring node was conditional, depending on the requirements and importance of the location.

According to the hardware specification of various oil and gas pipeline monitoring equipment, sensing nodes can remain connected to each other for their lifetime once they are installed and powered up. It is important to note that all the nodes are energy constrained and need unlimited power to support their task over the span of several years. In order to save on battery life, the sensing devices are configured to communicate with a minimum delay of 6 sec and 60 min at maximum. In order to save on battery lifetime, it is possible to decrease the wakeup time of sensing nodes and data transmission rate.

### 5.6. Energy Efficiency and Lifetime Concerns

Continuous condition monitoring requires a continuous, uninterrupted power supply. Wireless transceivers utilize most of the battery in a sensor node. Hence, increased battery life and optimal utilization of the power is of critical value especially for under water and buried pipelines. The commercially available sensor nodes usually provide a discrete, limited set of tunable power levels making dynamic adaptation less beneficial. The communication itself carried out by the sensor nodes in case of pipeline monitoring can be either periodic or event-based. More intelligent pipeline monitoring schemes use aggregated sensing [31] where the only primary sensing mechanism runs periodically, triggering other mechanisms and waking up nodes from sleep only when anomalies are detected. Some schemes incorporate intelligent node algorithms which perform correlation of the recently sensed data with the previous reading and transmit the reading only when the readings differ from the normal measurements [70]. A vibration energy harvester utilizes the energy available in kinetic form and converts it into electrical energy, usable for the sensor nodes. Underground energy scavenging can be done by converting seismic vibrations or thermal gradients to usable energy forms [29,30]. Electric power can also be generated using turbines embedded in the pipelines.

### 5.7. Remote Infrastructure Security Concerns

In the earlier wireless sensor network deployments, most networks were deployed without any security measures including the early implementation of the famous ZigBee protocol for sensing plane. The scenario changed and security measures were introduced at different layers when a multi-platform interoperability demonstration failed drastically since they interpreted the basic command code from a completely different network that was actually meant for the coordinator node in order to change channels, hence completely initiating a blackout in the system. At earlier stages, there were no measures for the sensor network to determine whether the message belonged to their own network or some other network. The basis for the failure was the requirement of an authentication mechanism, which led the system to interpret any type of message that was intrinsically relevant to the sensor domain [71].

When deploying wireless sensor networks in industry, particularly related to industrial automation applications, the consequence of architectural failures will be more than a mere loss of sensing information. With faulty, inaccurate or altered information being delivered to the control station, the system is potentially vulnerable to physical damage. For example, a sensor transmitting data to the valve controller or a direct motor informing it about the system speed requirements or component processing levels being too high could cause reversible damage to the whole industrial setup. Even in practical terms, a failed security aspect in the developed system or a revelation of weakness in the deployed sensor network could result in a loss of engineering power.

For a standalone secure network, security requirements need to be fulfilled at the protocol as well as system implementation and deployment level. Some examples of well-developed security protocols for sensor planes include the WirelessHART and ISA100.11a protocols related to industrial automation. The ZigBee Smart Energy related protocols have also undergone extensive review by security experts and numerous implantation tests. In particular, the WirelessHART protocol is the key enabling technology for most of the sensor network deployments around the world that are potential candidates for security concerns. The end users of the securely deployed networks put trust in the application in terms of provisioning processing control information reliably and in confidence between two authenticated sensor nodes [72]. In CPSN industrial applications, as new protocols emerge, particularly related to Internet of Things, the security measures need to be compatible with the IP communication procedures [73].

Once CPSN is set up, the realm of security concerns changes drastically. All such concerns need to be addressed with thorough understanding, assessment and a defense mechanism (Table 6). The major challenges and security related problems arise due to the decentralized nature and heterogeneity of protocol and hardware used to interconnect devices. The physical interactions between devices over an un-trusted environment open up potential vulnerabilities. Such issues are a major concern for CPS based on sensor networks with strict privacy and resource constraints.

**Table 6:** Cyber physical sensor network infrastructure-related security concerns.

Dimension	Parameters
Routing	Black Hole Attack, Hello Flood Attack, Worm Hole Attack, Denial of Service, Sybil Attack, Counterfeit Routing Selective Forwarding, Sink Hole Attack, Flood Attack, Acknowledgement Spoofing
Localization	Routing Misuse, Inaccurate Distance Measure
Transmission Modes	Broadcast Attacks, Multicast Attacks
Transport Layer	End-to-End Connection Disruption, Node Exhaustion, Network Link Exhaustion
Node Position Attacks	End Node Software Attack, End Node Hardware Attack, False Data Injection in Aggregator Cluster Role Destruction, False Relay Data Route
Covert Service Disruption Attack	Service Disruption, Service Misuse, Service QoS Loss
In-Network Security	Technical Failure, Employee Issue, Network Negligence, Internet Connection Concerns Deployment Area Access, Uncontrollable Circumstances
Consequences	Loss of Network Control, Loss of Node Control, Inaccurate Information Interoperability Failure, Loss of Engineering Effort

To assess the level of security implementation for a CPSn oriented system, diverse security goals need to be defined with major attacker models developed in relation to system complexity. The security metrics could be used to provide a scale of implementation with a holistic view of the complete system taking into account external vulnerabilities. Finally, defensive mechanisms for cyber systems normally must ensure continuous interaction between physical devices to highlight a clear gap between the role of attacker and defender [74]. These interactions could be complex enough depending upon the system distribution and environmental reliability.

## 6. Future Challenges and Open Research Issues

The energy efficiency in CPSN is one of the prime issues researchers have been working on for years. In order for sensing planes to perform their monitoring tasks for longer periods of time and due to the nature of their sensing in harsh environments, battery replacement is not an easy task and sometimes even impossible [75]. For extending network lifetime, much research work has been done on MAC protocols and routing in addition to energy harvesting.

MAC protocols provide efficient resource sharing and contribute in saving power by utilizing the node’s hardware only when it is needed. Major causes for energy consumption in wireless nodes are due to collision of transmitted packets, overhearing of transmitted packets, packet overheads and idle listening of the radios [8,9]. Numerous MAC protocols have been proposed but only a few manage to bear the harsh physical environment for sensing applications. Some of the widely used MAC protocols for WSN are SMAC, TMAC and BMAC [2]; however, the need for efficient MACs for pipeline monitoring applications with linear and hierarchical layouts is still an open issue. An optimum and well-designed antenna use can not only improve the energy efficiency of the system and transmission range but also provide reliable communication making the sensing node small enough to fit inside the sensing environment [11].

Wireless sensor nodes consume power in data sharing, processing, transmission/reception and in data routing. Routing protocol needs to be simple by reducing computational complexity and power efficiency in order to help increase the network lifetime [1]. There is a need for such cross layer efficient routing algorithms, particularly in different pipeline monitoring applications. Some initial work has been done in determining an optimum routing protocol [76,77] but still extensive efforts are required in this field. Reliability and robustness complements each other, reliability being the most desired aspect of the sensor system in order to perform and maintain its functions in normal as well as hostile environments [77], while robustness enables the system to handle errors during execution.

Middleware offers the ability to assimilate and reprocess software components on demand and help abstract the dissemination and heterogeneity of the underlying computing environment and services. It also supports the addition of non-functional values such as interoperability, load balancing, scalability, reliability, availability, usability, extensibility, manageability, reusability, services discovery, Quality of Service (QoS), stability, efficiency and security [21,22,51]. To address the design and implementation issues related to CPSN applications, a new approach to integrate the middleware layer has been proposed in [78]. The middleware is present between an operating system and the application layer in a sensor node. It can be divided into many sub-middleware functions some of which include time synchronization, location detection, battery-power control and networking [25]. In traditional computing devices, operating systems are well established, but for sensor nodes the applications are executed on bare hardware without a separate operating system [79]. Hence, the identification and implementation of appropriate operating systems and middleware in CPSN is still a research focus [51,80]. Research could be initiated on developing different reliable and robust sensor nodes for diverse types of pipelines with oil and gas monitoring as summarized in Table 7.

**Table 7:** Summary of CPSN architectures for infrastructure monitoring.

Reference	Technique Specifics	WSN-CPS Integration	Intelligent Data Processing	QoS	Mobility	Cross Layer	Level of Control	Localization	IP-Based	Energy Efficiency	Applications
[18]	Multi Application Quality Monitoring	Yes, Middle Level Resource Sharing	Yes, Dedicated Computing Resources	Quality Of Monitoring	No	Yes, Multi-Application Allocation and Deployment	High	Yes	Yes	Not, Available	Multi Application
[25]	General Survey	Yes	Yes	Event Handling, Network Coverage	No	Yes (Domain Intelligence)	High	Yes (Knowledge Mining)	No	Low	Intelligent Transportation, Social Networking
[44]	Cloud Software as a Service	Yes	Yes	Yes, IP Based	Yes, Software Level	Yes, Implementat-ion Abstraction	Yes, Software Based	Yes	Yes	Not, Available	Network Monitoring, IP Based Services
[57]	Unmanned Vehicle Detection	Yes Integrated	Yes	Software Vision System	Yes, Vehicle Navigation	Yes	Very High	Yes, GPS–0.67 m Accuracy	No	Energy Controller	Vehicle Trajectory
[58]	Structure Condition Reporting	Yes	Multi level Computing	Flexible Network Operation, Multi-resolution Feature	No	Flexible Software Integration	High	Yes (Damage Localization)	No	Yes	Structural Health Monitoring
[60]	Locating Structure Damages	Partial	Yes	64.8% Latency Reduction	No	No	Limited	Yes, DLAC Algorithm	No, Decentrali-Zed	69.5% Less Power Usage	Infrastructure Damage Localization
[61]	Internet of Things	Limited	Yes	Lighter Algorithms	Yes	Yes, Software Based	Medium	Yes	Yes	Yes	Energy Monitoring, Social Networking
[66]	Sensor Node Localization	Limited	Yes	Service Application Based	Yes	Limited	High	Range Free, Proximity, Multi hop	Yes	Yes	Cyber Transportation, Social Networking
[70]	Soil Moisture Monitoring	Yes, Full	Yes, Collection, Management, Visualization	Confirm with Sensor Web Standard	Yes	Yes	Yes, Unification	Yes	Yes	Not, Available	Environment Monitoring
[81]	IEEE 802.15.4 Evaluation	Yes	Yes	Effective Data, Packet Loss Rate, End-to-End Delay	Yes	Yes	No	Yes	Yes	Yes	General Service Application (Video, Voice)
[82]	Sensor Grid	Yes	Yes, Analysis, Forecast, Data Repository	Real-time Data	No	No	No	Yes	No	No	Weather Forecast
[83]	Packet Loss Control with Actuation	Yes, Integrated	Yes, Prediction	Packet Loss, Mixed Traffic Reliability	No	Yes, Platform Heterogeneity	Yes, Actuation	Yes, Dynamic Network Topology	Yes	Yes, Resource Constrained	General
[84]	Plume Detection DITSCN	Yes	Yes, 3-D Data Analysis, Storage and Computational Resources	Medium	Yes	Yes	Medium	Yes, Movement and Location Tracking	No	No	Plume Detection and Tracking Biological Application
[85]	Aggregate Load Modelling Using Data Mining	Yes	Mathematical Relation and Analysis	Highly Interactive Protocols	Yes	Yes, High	High	Distributed Algorithm	Yes	Yes	Complex Electric Power System Monitoring, Distributed Sensing and Control
[86]	Cellular Networking	Yes	Yes, Data (Alerts) Pre-processing	Wide Coverage Real-time	Yes	No	Software Alerts	Limited Cellular Tracking	Yes	Not, Available	Vehicular Monitoring
[87]	Pipeline Leakage Detection -REMONG	Yes, Cellular and Internet	Wavelet Signal Processing And Learning Algorithms	Linear Routing With Feedback And Link Status	No	Yes	Software Alerts, Valve Actuation	Yes, Leakage Between Routers	Yes	Node Transmission Power Control	Oil and Gas Pipeline Monitoring

The radio link between low-power sensor networks is extremely unreliable due to harsh environments in which they are deployed. In the case of pipeline monitoring, the environment gets even tougher since oil and gas pipelines in desert areas have to bear heat and humidity while pipeline under sea bears huge water pressure. Underground pipelines also present challenges in communication since radio waves at ground level may have to deal with multiple paths. Hence, a reliable communication model for a secure and energy efficient communication between wireless nodes is one of the main research areas for current and future efforts [17]. The need for new, efficient and small sensors for monitoring leaks and quality of fluids like water, oil and gas is increasing. New sensing techniques based on novel approaches for precision and physical phenomenon detection will be able to enhance the monitoring systems’ reliability and robustness by eliminating the sensing errors and increasing efficiency [88,89].

**Figure 13:**
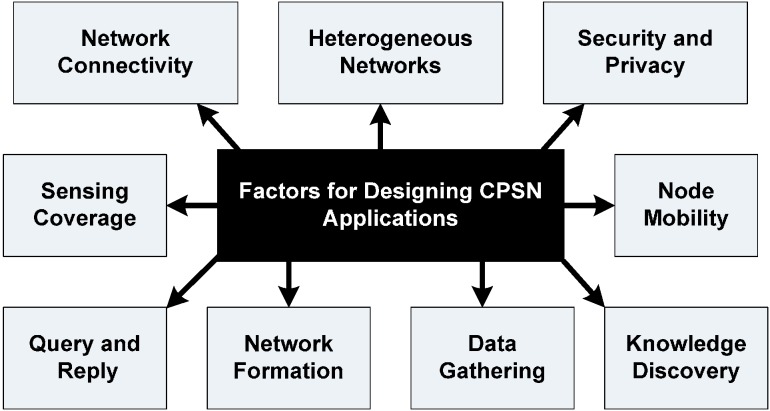
Relevant factors for designing CPSN applications.

The sensing nodes should be able to reconfigure for sensing at different sampling rates and adapt to different conditions and scenarios in order to support the application and ease of monitoring. Research to develop adaptable and configurable wireless nodes for the pipeline monitoring systems is required with growing complexity of sensing information and reliability. It is also important for the system to be scalable with future improvements and enhancements [90]. Finally, scalability for pipeline monitoring systems is a critical issue due to harsh sensing environments, piping structures and difficult terrains. Hence to summarize, major design factors for CPSN applications need to consider physical sensor network type, application security, methods to convert raw data into meaningful information and technology to support seamless data flow (Figure 13).

## 7. Conclusions

Cyber Physical Systems (CPSs) have taken their roots from mergers between computational and physical components of systems. CPS has played a critical role in a number of underlying domains namely healthcare, manufacturing, energy, transportation, aerospace and industrial infrastructure based conditional monitoring. A complete transformation of human-to-human, human-to-machine and machine-to-machine interactions is expected with the introduction of virtualization [90]. While previously, much of the research activities focused on Mobile Ad Hoc Networks and Wireless Sensor Networks (WSNs), more recently, a changing trend has been to benefit from the physical and virtual environment synergy provided by CPS to perform the conventional activities of WSN more reliably and with ease. In this work, we have reviewed the applications of a Cyber Physical Sensor Network (CPSN) environment from a reliability perspective and demonstrate how the physical information collected from different sensing planes be exploited to abridge the cyber space and real world. We also identified the challenges and architecture design issues of CPSN. The techniques and parameters that still need to be addressed for seamless integration of cyber and sensing domains with QoS and the current measures adopted have also been summarized.

While the sensing plane focuses more on the designs for sensing, data-retrieving, event-handling, communication, and coverage problems, the cyber plane focuses on the development of cross-layered and cross domain intelligence from multiple sensing environments and the interactions between the virtual world and the physical world [91]. A CPSN application is expected to provide a bridge between multiple remote WSNs and invoke actuation based on inference from the sensed information [92]. A lot of successful vehicle- and mobile phone-based CPSN services have been developed over time [93]. Data from such applications may be expected to be of continuous form at a very large volume, so storing, processing, and then intelligent interpretation of it in real-time is essential. Important factors for the success of CPSN include management of cross-domain sensor related data, embedded and mobile sensing technologies and applications, elastic computing and storage related technologies with integrated privacy and security designs. We have also reviewed different platforms for oil and gas monitoring, navigation and rescue services, social networking and gaming with related challenges in these systems summarized here. This summary of CPSN is expected to stimulate an interested reader with current technological developments and the expected features of future sensing networks over the Cloud.
